# Molecular dynamics investigation of charging process in polyelectrolyte-based supercapacitors

**DOI:** 10.1038/s41598-022-04837-4

**Published:** 2022-01-20

**Authors:** Nasrin Eyvazi, Morad Biagooi, SeyedEhsan Nedaaee Oskoee

**Affiliations:** 1 Department of Physics, Institute for Advanced Studies in Basic Sciences (IASBS), Zanjan, Iran; 2 Research Center for Basic Sciences & Modern Technologies (RBST), Institute for Advanced Studies in Basic Sciences (IASBS), Zanjan, Iran

**Keywords:** Chemistry, Energy science and technology, Physics

## Abstract

Supercapacitors are one of the technologically impressive types of energy storage devices that are supposed to fill the gap between chemical batteries and dielectric capacitors in terms of power and energy density. Many kinds of materials have been investigated to be used as supercapacitors’ electrolytes to overcome the known limitations of them. The properties of polymer-based electrolytes show a promising way to defeat some of these limitations. In this paper, a simplified model of polymer-based electrolytes between two electrodes is numerically investigated using the Molecular Dynamics simulation. The simulations are conducted for three different Bjerrum lengths and a typical range of applied voltages. The results showed a higher differential capacitance compared to the cases using ionic-liquid electrolytes. Our investigations indicate a rich domain in molecular behaviors of polymer-based electrolytes that should be considered in future supercapacitors.

## Introduction

Utilizing fossil fuels to produce energy has some disadvantages, such as a limited amount of resources as well as environmental pollution^1^. In addition, a world-wide replacement of the limited energy sources with unlimited and clean ones like solar energy requires existence of durable and inexpensive energy storage devices^2,3^. Supercapacitors, which are also known as electric double-layer capacitors (EDLCs), are ideal reversible electrochemical energy storage devices^3^ and are one of the main candidates for substituting ordinary batteries. Due to their advantages like higher power density, rapid charging/discharging cycles, long cycle life, and the high specific capacitance EDLCs are used in a variety range of the applications such as portable electronics, hybrid electric vehicle-based green transportation, backup energy sources, medical utilities, and spaceships^1,4–8^. Despite the high electric power of supercapacitors, their stored energies are relatively low^9^, therefore they can not be replacement for chemical batteries yet. To deal with this issue, subnanometer porous electrodes like carbon-based materials with high specific surface area are used in creating EDLCs^8^. The high capacitance of EDLCs comes from the formation of an electric double-layer at the electrode/electrolyte interface^6,8,10^. When a potential difference is applied between the electrodes, the separation of opposite charges occurs. The charged electrodes adsorb the opposite-charged electrolyte ions without any chemical reactions resulting in a long life for charge/discharge cycles and rapid charging rate compared to the batteries^11,12^.

The efficiency of EDLCs significantly depends on the electrode and electrolyte structures^4^. In an earlier version of supercapacitors, liquid electrolytes containing aqueous, organic materials, and Ionic-Liquid (IL) are utilized^4,8,13^. EDLCs with such electrolytes are associated with problems like leakage of electrolytes^4,6,7^, bulky design^6,7^, limited transportability^7^, and pollution of the environment^4^. As a consequence of these problems, demands for considering new materials for electrolyte arises. In this case, polymer-based electrolytes have attracted considerable attention^7^. The advantages of polymer-based electrolytes include high performance, light weight^14^, mechanical stability, safety, simple processing^15^, high ionic conductivity, wide operation temperature, wide electrochemical window, absence of leakage^4^ and corrosion problems^10^. Polyelectrolytes belong to a particular class of polymer-based electrolytes in which their repeating units have permanent charges. They can be divided as polycations, polyanions, and polyzwitterions where polycations (polyanions) have a cation (anions) in the backbone’s part of their monomer unit. In contrast polyzwitterions have both cation and anion groups^16,17^. The polyelectrolytes which are used in experimental investigations are usually chosen from the following set: polystyrene sulfonate, polyacrylic, polymethacrylic acids and their salts, DNA, other polyacids and polybases^18^, linear polymers such as polyacrylonitrile (PAN), poly(ethylene oxide) (PEO) and poly(vinylidene fluoride-co-hexafluoropropylene) (PVDF-HFP)^10^.

Polyelectrolyte-based supercapacitors have not been numerically investigated yet; however, because of the complex structure, understanding the details of the charging process is not possible without the help of simulation. In this work, we tried to numerically investigate these types of supercapacitors using extensive molecular dynamics simulation. To do that, we used a simple model of the charged polymer as the polyelectrolyte. Furthermore, conductive electrodes were modeled as a boundary condition with a constant potential. Our purpose is to investigate the properties of polyelectrolyte-based supercapacitors and compare them with those containing IL electrolytes.

## Results and discussion

Using the molecular dynamics package of CAVIAR^19^, we carried out the simulations of systems containing asymmetric electrodes and two kinds of polymers with the opposite charges as electrolytes. The electrodes are conductive boundaries which the potential differences are applied between them. The charged polymers were modeled as simple bead-spring chains connected with harmonic bond potential. To investigate the effect of electrostatic interaction strengths of the solution, the concept of Bjerrum length was used.

**Figure 1:**
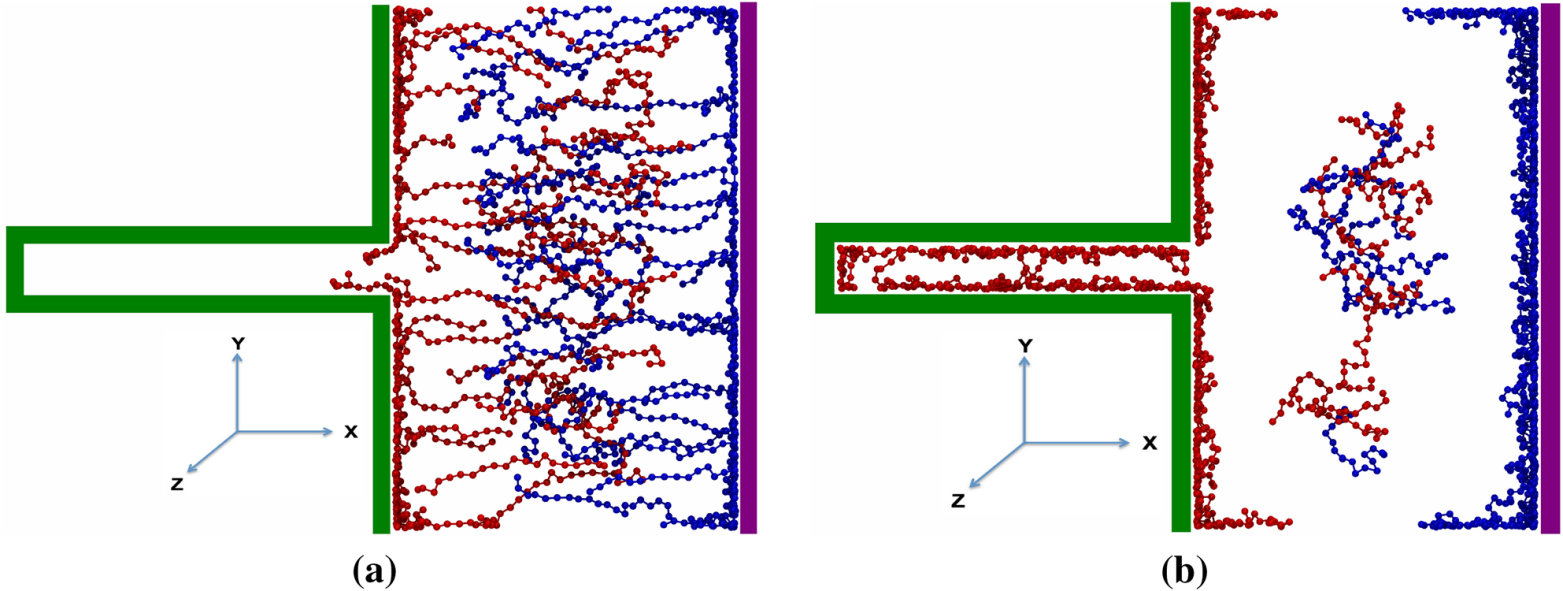
Two snapshots of the system at different times; (**a**) at early stages of the simulation, and (**b**) when charged polymers separation occurred.

The simulations were performed for 3 different values of the Bjerrum length: $\lambda _{B}$ = 0.72, 2.4, 5.6 nm. According to Eq. (6), the Bjerrum length is inversely related to the relative permittivity of materials. For $\lambda _{B} = 0.72~\mathrm{{nm}}$ at the temperature 300 K, the $\epsilon$ = 78 F/m, which corresponds to the relative permittivity of water. For $\lambda _{B} = 2.4$ nm, the well-known relative permittivity in polyelectrolyte solution is 22.5 F/m (the relative permittivity of Ethanol)^16,20^. The $\lambda _{B} = 5.6$ nm at temperature 300 K corresponds to $\epsilon =10$ F/m (the relative permittivity of methylene choloride) that was used previously in the article^21^ for IL-based supercapacitors. It is compatible with ionic liquid solution. Figure 1 is a snapshot of the simulated system at two different time steps before (Fig. 1a) and after (Fig. 1b) separation opposite charged polymers.

### Electrode charge fluctuations and differential capacitance

**Figure 2:**
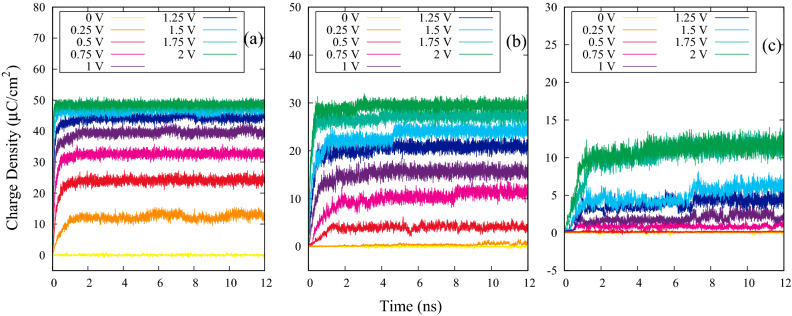
Time evolution of induced charge density on the slit-pore electrode with N = 20; for (**a**) $\lambda _{B}=0.72$ nm , (**b**) $\lambda _{B}=2.4$ nm, and (**c**) $\lambda _{B}=5.6$ nm.

Figure 2 displays the time evolution of induced charge density on the slit-pore electrode for different $\lambda _{B}$. As can be seen from Fig. 2, the induced charge density on the electrode increases with increasing voltage. For larger $\lambda _{B}$, the system reaches equilibrium later. Also, because increasing the $\lambda _{B}$ increases the electrostatic strength of the solution, polymers with opposite charges attract each other more strongly and it becomes difficult to separate them. Therefore, the induced charge density on the electrode is reduced. In addition, this figure shows the equilibrium of the system.

**Figure 3:**
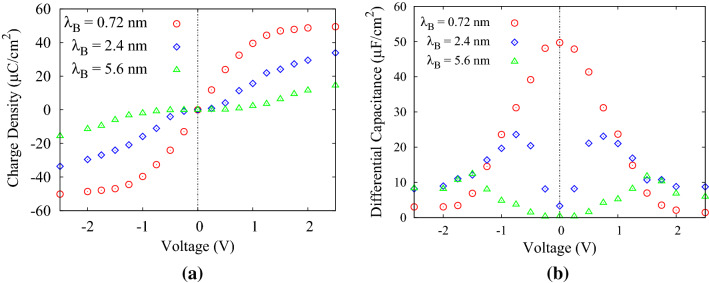
(**a**) Mean induced charge density, and (**b**) differential capacitance; as a function of applied potentials for N = 20.

Figure 3a is the plot of mean induced charge density after the system is equilibrated as a function of potential differences between the electrodes. According to this figure, the induced charge arises with increasing the applied potential difference between the electrodes. In contrast, the electrostatic strength of the environment has a direct relation with $\lambda _{B}$, as a result, increases the numeric value of $\lambda _{B}$ make electrical interaction more strong. Growth of the attraction force results in the polymers with opposite charges entangling, making them difficult to separate. In this case, complete separation does not occur and there are still some charges in the bulk region even when the system reaches equilibrium. Therefore, increasing the $\lambda _{B}$ not only expands the equilibrium time but also reduces the amount of induced charges on the electrodes.

In ideal dielectric capacitors, the induced surface charge on the electrodes linearly in enlarged by enhancing the applied potential difference between the electrodes. Hence, according to the definition, the capacitance of such capacitors is constant for any applied voltage. In contrast, the capacitance in EDLCs is a function of applied voltage; therefore, Differential Capacitance (DC) is defined and used to describe the EDLCs’ capacitance properties^21^. Indeed, DC is defined as a quantitative measure of the response of the electric double-layer structure to a change in the charge density of the electrodes^22^. The specific DC of the EDLCs is defined as^23^

1 $$\begin{aligned} C_d(V)=\frac{d\sigma }{dV}, \end{aligned}$$

where $\sigma$ is the induced surface charge density and *V* is the potential drop between the bulk of electrolyte and the electrode surface^23^. Therefore, according to Eq. (1), DC is obtained through the numerical derivative of the graph of Fig. 3a.

Figure 3b is the DC plot as a function of the potential difference between the electrodes for 3 different values of $\lambda _{B}$. This plot which is obviously imitating the ion concentration behavior near the electrodes, has been obtained by numerical differentiation of induced charge density using central difference derivation method. For the $\lambda _{B}$ = 0.72 nm, the DC curve is bell-shaped. According to a mean-field theory developed by Kornyshev^23^, for high ion concentration near the electrode such as $\lambda _{B}$ = 0.72 nm in our case, the maximum of DC occurs at zero potential difference (Potential of Zero Charge (PZC))^24^. Therefore, as the potential increases, capacitance decreases, resulting in a bell-shaped curve^21,23^. In contrast, for the low ions density near the electrode and at low voltages, $C_d(V)$ follows the U-shaped model with a $V^{-1/2}$ reduction at higher voltage which was predicted by Kornyshev theory. In this case, the resulting curve is a camel-shaped curve^21^. Figure 3b presents the camel-shaped curve for $\lambda _{B}$ = 2.4 and 5.6 nm due to their low ion concentration at the electrode-electrolyte interface compared to the $\lambda _{B}$ = 0.72 nm. Moreover, the maximum of the DC decreases by increasing the $\lambda _{B}$, due to the reduction of the induced charges on the electrodes.

**Figure 4:**
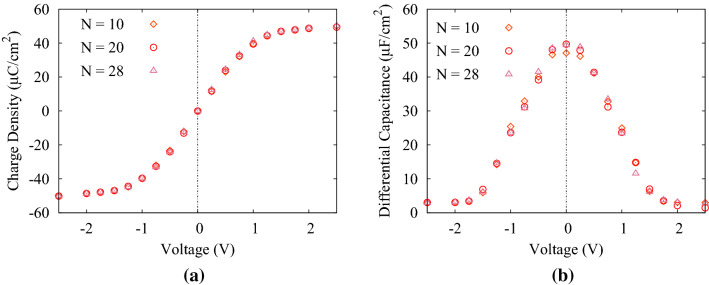
(**a**) Mean induced charge density, and (**b**) differential capacitance; as a function of applied potentials with $\lambda _{B}=0.72$ nm for 3 different N.

For investigating the effect of polyelectrolytes length, we plotted the charge density and the DC for 3 different values of N. The simulations performed for $\lambda _{B}=0.72$ nm. The mean charge density and the DC have been exhibited in Fig. 4a,b for N = 10, 20, 28. By these figures, it is shown that the results are independent from the number of monomers and therefore the charge density and the DC are identical for all 3 different N.

### The structure of ion layers

**Figure 5:**
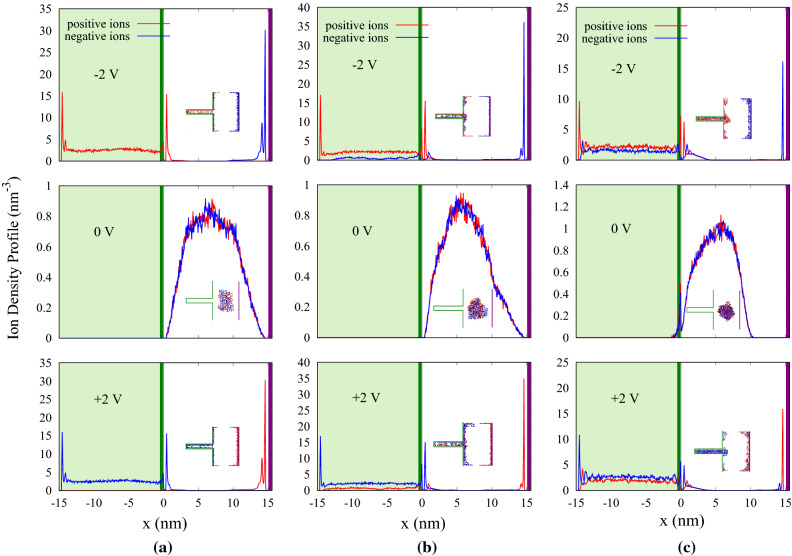
Ion density profiles for three different voltages applied between electrodes along x position for (**a**) $\lambda _{B} = 0.72$ nm, (**b**) $\lambda _{B} = 2.4$ nm, and (**c**) $\lambda _{B} = 5.6$ nm. The green and the purple lines represent the position of the electrodes. Light green shadow displays inside the pore. Inset is a snapshot of the system at last stage of the simulation.

To obtain more information about the structure of polyelectrolytes at the electrode boundaries, the ion density profiles along the x-axis are plotted (Fig. 5a–c). As shown in these figures, at zero voltage, the polyelectrolytes in the bulk region accumulate due to the electrostatic interactions between charged polymers. In the absence of the applied potential difference, depending on the numeric value of $\lambda _{B}$, oppositely charged chain systems tend to reach three different states; globular states, extended chains and pearl-necklace-like sequences^25^. For larger interaction parameters, the chains start to collapse and aggregate, fold into densely packed structures. In our simulations there is evidently an attractive interaction in the system present, which results in rise of a compact structure constrained to a small region in space. Applying a positive voltage difference (2 V), an electric field is formed along the x-axis which causes the positively charged polymers to be deflected towards the higher voltage porous electrode. For lower Bjerrum length ($\lambda _{B} = 0.72$ nm), the applied electrical field is stronger than electrical interaction between polymers resulting in complete separation (Fig. 5a). As a consequence, only one type of polymer enters the pore. In comparison, as $\lambda _{B}$ increases, internal electrical interaction between oppositely charged polymers becomes stronger. this in turn leads to a partial separation in polymers; therefore, polymers with opposite charges can be found inside the pore as well as near the electrodes (Fig. 5b,c). The charges exhibit a layering structure near the pore opening and closing, while in the middle of the pore, they are distributed homogeneously. The case will be vice versa when the polarity of applied voltage changes.

Typically, electrolytes can dissociate into positive and negative ions. With the above explanation, it can be concluded that dissociating of ions occurs for nonzero voltages. The energy required to separate an ion pair varies inversely with the relative permittivity (dielectric constant), and, therefore, dissociation into ions with opposite charges happens only in solvents with large dielectric constants (small $\lambda _{B}$). In addition to electrolytes, the dissociation of materials like water under an external electric field is of great current interest and has been broadly investigate^26^.

### Polymers conformation

**Figure 6:**
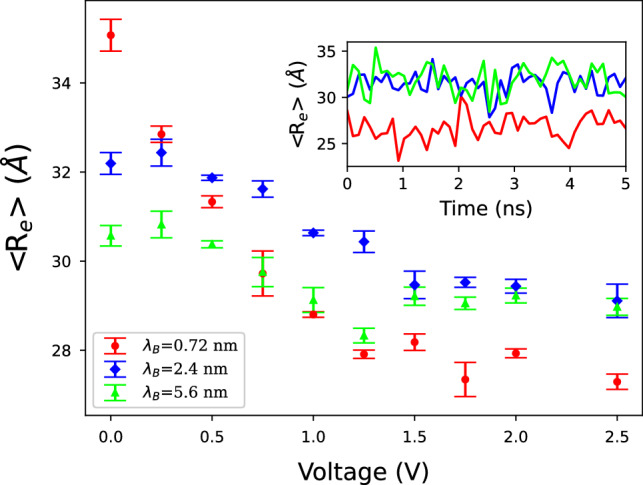
Mean end-to-end distance of polymers ensemble for different $\lambda _{B}$ with N = 20. Inset is the time evolution of the $\langle R_e\rangle$.

Different conformations of charged polymers during simulation time are analyzed. To do that the mean square end-to-end distance is computed for 3 different $\lambda _{B}$ (Fig. 6). This quantity is calculated according to the following equation^27^:

2 $$\begin{aligned}\langle R_{e}^{2}\rangle = \langle \left( \mathbf{r} _{N}-\mathbf{r} _{1}\right) ^{2}\rangle, \end{aligned}$$

where $\mathbf{r} _{N}$ is the position of the last monomer and $\mathbf{r} _{1}$ belongs to the first one. The following points can be deduced from this figure:

1. For $\Delta V=0~{\text{V}}$, $\langle R_{e}\rangle$ decreases when $\lambda _{B}$ increases. In the absence of the applied electric field, internal electrical interactions between polymers with opposite charges yields to polymer clustering in the bulk region. The longer the $\lambda _{B}$, the stronger electrostatic interaction. As a result, it ends up having more entanglement of polymers. Increasing the entanglement of polymers can be seen as a reduction of $R_e$.
2. As the voltage increases, the $\langle R_e\rangle$ decreases. The speed of reduction is more for polymers with smaller $\lambda _{B}$, and at the sufficiently large voltage difference, the order of $\langle R_e\rangle$s is opposite compare with the case of $\Delta V=0$ V. The origin of this difference comes form the fact that at high $\Delta V$ polymers with opposite charges separated completely from each other and stick to electrode walls which contain surface charges with opposite sign. In this case, the attractive force between the electrode surface charges and the polyelectrolytes near the electrode boundaries causes polymers to shrink. Polymers with smaller $\lambda _{B}$ have weaker repelling interaction, so they shrink more around boundaries surface charges, which contribute to smaller $R_e$.
3. The inset plot in the figure shows the time evolution of $\langle R_e\rangle$ for $\Delta V=2$ V. In this case, for a longer $\lambda _{B}$, the repulsive force between the monomers with the same charge is increased as well. consequently, they repel each other more intensely. Express differently, the longer the $\lambda _{B}$, the more stretching the polymers. The small size of the system causes fluctuations in conformation.

### Comparison of IL-based with polyelectrolyte-based supercapacitors

**Figure 7:**
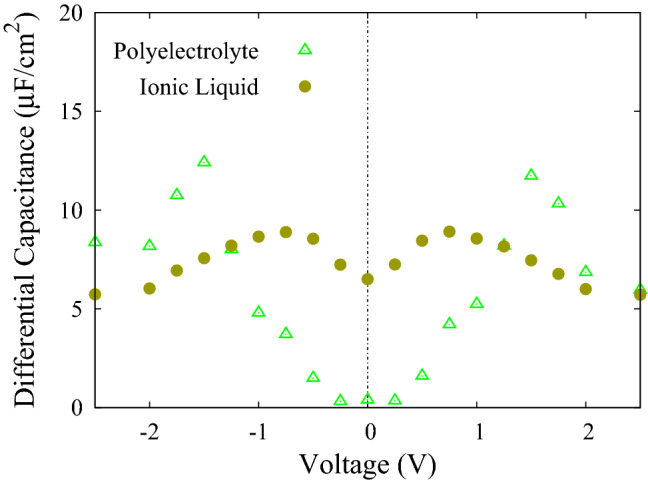
A comparison between differential capacitance for polyelectrolyte-based supercapacitor and IL-based supercapacitor with $\lambda _{B}$ = 5.6 nm.

Comparing the properties of two types of supercapacitors with different electrolytes containing ILs and polyelectrolytes can illustrate some possible differences between them. To make comparison meaningful, both electrolytes should have the same Bjerrum length. For this reason, based on what has been discussed earlier in this manuscript, the IL simulations for $\lambda _{B} = 5.6$ nm are conducted. Figure 7 is a comparison between DCs of IL-based and polyelectrolyte-based supercapacitors.

As shown in Fig. 7, there is a big gap at the zero potential between the values of DC. Since both of the simulations are conducted at the similar $\lambda _{B}$ value, this gap comes from the difference between amount of forces needed to separate entangled polymers and ILs ions; it is easier to separate ions of IL and move them toward the electrodes than charged polymers twisted to each other. As the voltage is increased, however, the applied potential can separate some of the entangled chains. Because a chain contains more than one charged particle ($N = 20$ in our case) a sharp growth in the DC curve of polyelectrolytes is observed.

According to this Figure, the maximum of DC for ionic liquids occurs at 0.75 V. At larger voltages, the DC decreases and eventually reaches saturation. In contrast, the maximum of DC for polyelectrolytes occurs at 1.5 V, and also for larger voltages, the DC of polyelectrolytes is higher than ionic liquids. Therefore, according to $E(V) = \int V~C_d(V)~dV$^12^, it is expected that the stored energy in polyelectrolyte supercapacitors is more than ionic liquids. This result can have a significant effect on improving the efficiency of supercapacitors.

**Figure 8:**
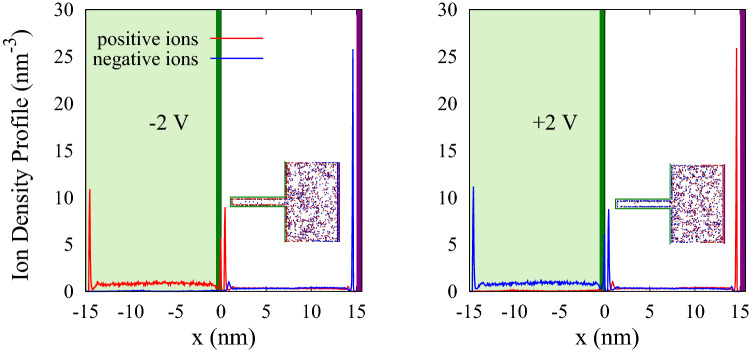
Ion density profiles for two different voltages applied between electrodes along x position for IL-based supercapacitors. The green and the purple lines represent the position of the electrodes. Light green shadow displays inside the pore. Inset is a snapshot of the system at last stage of the simulation.

Figure 8 shows the ion density profile for the system containing ILs. By comparing this figure with Fig. 5c, we find that in the case of IL-based supercapacitors, both positive and negative ions exist in the bulk region. In polyelectrolyte-based supercapacitors, however, the polymers are all concentrated near the electrodes, and there is no charge in the bulk region.

## Conclusions

In summary, the simulations showed the differences between using ionic liquids and polyelectrolytes as the construction unit of the supercapacitor electrolyte. The simulated polymer model is similar to the single-particle IL model^9,13^ with an additional harmonic force that links the monomers together. The links between the similarly charged particles make them spatially constrained to their respective chain. It means that the thermal fluctuations will be averaged for a polymer chain, and the center of mass of each chain will be more thermally stable than of a single IL particle. Also, the monomers are forced to move in groups with a net charge proportional to the degree of polymerization, meaning that the effects of the external force due to the electrodes on each monomer are summed for a chain, which makes a chain move toward the electrodes easier than free ions.

## Methods

### The models and system parameters

The molecular dynamics simulations of systems contain two kinds of polymers with the opposite charges (polycations and polyanions) were performed by using a CAVIAR software package^19^. In these simulations, the simplest representation for charged polymers was described by the coarse-grained bead-spring model^25,28^. With N as the degree of polymerization, N beads (monomers) were connected to each other by N-1 harmonic springs. These harmonic bonds were described by the following potential:

3 $$\begin{aligned} U_{b} = K \, \sum _{i=1}^{N} \, \left( |\mathbf{r} _{i}-\mathbf{r} _{i-1}|-l_{0}\right) ^{2}, \end{aligned}$$

where $l_{0}$ is the equilibrium bond length between the monomers. To limit the bond length fluctuations, a considerable value was selected for the energy constant^25,28^ $K = 2500~\varepsilon / \sigma ^{2}$, where $\varepsilon$ and $\sigma$ are the LJ potential parameters. Each beadlike monomer has a diameter equal to $\sigma =5$ Å. All particles in the system interact through a truncated-shifted LJ potential^29^:

4 $$\begin{aligned} U_{LJ} = \left\{ \begin{array}{lr} 4 \varepsilon \left[ \left( \frac{\sigma }{r}\right) ^{12} - \left( \frac{\sigma }{r}\right) ^{6} - \left( \frac{\sigma }{r_{cut}}\right) ^{12} + \left( \frac{\sigma }{r_{cut}}\right) ^{6}\right] &{} ; \quad r \le r_{cut} \\ 0 &{} ; \quad r \ge r_{cut} \end{array}, \right. \end{aligned}$$

where *r* is the relative distance between any two pairs of particles in the system. In our simulations the equilibrium bond length is set to $l_{0}=\sigma$ and the cutoff length is $r_{cut} = \root 6 \of {2}~\sigma$.

Moreover, the electrostatic interaction between each pair of two charged beadlike monomers was taken into account using the Coulomb law:

5 $$\begin{aligned} U_{C} = \sum _{i}\sum _{i<j}\frac{z_{i}z_{j}e^{2}}{4\pi \epsilon |\mathbf{r} _{i} - \mathbf{r} _{j}|}, \end{aligned}$$

where $\epsilon$ and *e* are the electric permittivity and the electric charge. The $z_{i} = \pm \,1$ shows the charge of each monomer. Here, we simulated polyelectrolytes in salt-free solution at different electrostatic interaction strengths. The strength of electrostatic interaction was given by the Bjerrum length:

6 $$\begin{aligned} \lambda _{B} = e^{2}/4\pi \epsilon k_{B}T, \end{aligned}$$

defined as the length scale at which the electrostatic interaction between two elementary charges *e* in the medium with dielectric permittivity $\epsilon$ is of the order of the thermal energy $k_{B}T$^27^.

All the simulations were performed at a constant temperature using the Langevin thermostat in which the system is coupled to an implicit solvent, acting as a thermal bath. Therefore, the equation of motion of the system was given by the Langevin relation:

7 $$\begin{aligned} m \ddot{\mathbf{r }}_{i} = -\nabla _{i} U\left( \{\ \mathbf{r} _{j}(t)\}\right) - \gamma m \dot{\mathbf{r }}_{i} + \mathbf{F} _{i}, \end{aligned}$$

where the first term describes the deterministic forces between monomers (the force acting on atom *i* due to the interaction potentials), and the last two terms implicitly consider the effect of the solvent by coupling the system to a Langevin thermostat^27^ which maintains a constant average temperature of the system. The parameter $\gamma$ is the friction coefficient and $\mathbf{F} _{i}$ is a Gaussian distributed random force with^27^:

8 $$\begin{aligned} \begin{array}{lr} \langle \mathbf{F} _{i}(t) \rangle = 0 \\ \langle \mathbf{F} _{i}(t) \mathbf{F} _{j}\left( t^{\prime }\right) \rangle = 6k_{B}Tm \gamma \delta _{ij} \delta \left( t-t^{\prime }\right) . \end{array} \end{aligned}$$

Our simulation box consists of 1680 particles where half of them are particles with positive charges and the remains are negative charged particles. The simulations were executed for N =10, 20, 28 spherical monomers connected by the harmonic stretching bond in each polyelectrolyte. Lennard–Jones (LJ) reduced unit was used to make the governing equation of motions dimensionless. Length is scaled with ion size $\tilde{l} = \sigma =5$ Å, mass unit is $\tilde{m} = 144$ g/mol, the energy unit is $\tilde{\varepsilon } = 1$ kJ/mol and the charge unit is $\tilde{q} = e$. Therefore, time unit is obtained $\tilde{t} = 2$ ps by applying $\tilde{t}=\sqrt{\dfrac{\tilde{m} \sigma ^{2}}{\tilde{\varepsilon }}}$. In addition, temperature scales as $\tilde{T}=\dfrac{\tilde{\varepsilon }}{k_B}=120.267$ K and voltage $\tilde{V}=\dfrac{\tilde{q}}{\tilde{\varepsilon }}=0.01036$ V.

The temperature was chosen as $k_{B}T = 2.5~ \varepsilon$ which corresponds to 300 K in real unit. The friction coefficient in the Langevin equation was set to $1/ \gamma = 1/\tilde{t}$ and the time step was $\Delta t = 0.001~\tilde{t}$ equal to 2 fs.

### The electrode modeling and potential difference between electrodes

**Figure 9:**
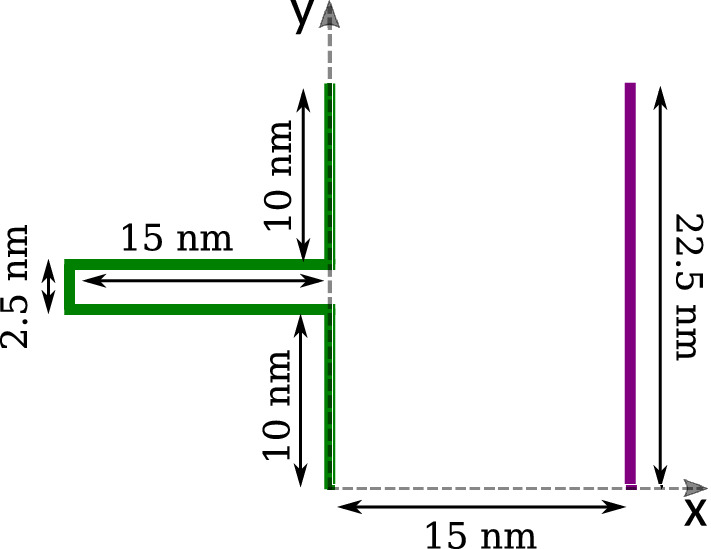
Asymmetric electrodes geometry: one flat electrode (purple) and one with slit-pore (green). The electrodes are modeled geometrically instead of using atoms.

We have investigated the behavior of the described system confined between two electrodes separated by a distance of 15 nm with single slit-pore geometry, as shown in Fig. 9. This is a simple model which is called a slit-pore model for simulating porous media, introduced by Breitsprecher et al.^9,11^. Recently, Biagooi et al.^13^ have used this geometry to simulate the IL-based supercapacitors. The pore size is often comparable of the bulk region; therefore the slit-pore length was set to be 15 nm like the bulk region, and its width was 2.5 nm (greater than twice the ion size) to allow the coverage of both pore walls^30^ and let unless two ions enter the pore.

The electrodes were built from carbon atoms with the following parameters: $\sigma _{C} = 3.37$ Å and $\varepsilon _{C} = 1$ kJ/mol. These are surfaces with the constant potential. Here, the constant potential was performed by using the Poisson to Laplace Transformation (PLT) method, which is recently developed in the CAVIAR package^19^. Periodic boundary condition applied in the XY plane and the long-range coulombic interaction performed using a 1D Ewald algorithm^31^ with $R_C=15~\sigma$ (smaller than half of the size box) as truncated electrostatic interaction distance. The system is simulated at 19 various potential difference between electrodes: 0, 0.25, 0.5, 0.75, 1, 1.25, 1.5, 1.75, 2, 2.5 V as well as their negative values. At first, each simulation is run to reach equilibrium; thereafter, data collection proceeded for about 10 ns.
